# An Interspecies Comparative Analysis of the Predicted Secretomes of the Necrotrophic Plant Pathogens *Sclerotinia sclerotiorum* and *Botrytis cinerea*

**DOI:** 10.1371/journal.pone.0130534

**Published:** 2015-06-24

**Authors:** Steph Heard, Neil A. Brown, Kim Hammond-Kosack

**Affiliations:** Plant Biology and Crop Science, Rothamsted Research, West Common, Harpenden, Hertfordshire, United Kingdom; The University of Wisconsin - Madison, UNITED STATES

## Abstract

Phytopathogenic fungi form intimate associations with host plant species and cause disease. To be successful, fungal pathogens communicate with a susceptible host through the secretion of proteinaceous effectors, hydrolytic enzymes and metabolites. *Sclerotinia sclerotiorum* and *Botrytis cinerea* are economically important necrotrophic fungal pathogens that cause disease on numerous crop species. Here, a powerful bioinformatics pipeline was used to predict the refined *S*. *sclerotiorum* and *B*. *cinerea* secretomes, identifying 432 and 499 proteins respectively. Analyses focusing on *S*. *sclerotiorum* revealed that 16% of the secretome encoding genes resided in small, sequence heterogeneous, gene clusters that were distributed over 13 of the 16 predicted chromosomes. Functional analyses highlighted the importance of plant cell hydrolysis, oxidation-reduction processes and the redox state to the *S*. *sclerotiorum* and *B*. *cinerea * secretomes and potentially host infection. Only 8% of the predicted proteins were distinct between the two secretomes. In contrast to *S*. *sclerotiorum*, the *B*. *cinerea* secretome lacked CFEM- or LysM-containing proteins. The 115 fungal and oomycete genome comparison identified 30 proteins specific to *S*. *sclerotiorum* and *B*. *cinerea*, plus 11 proteins specific to *S*. *sclerotiorum* and 32 proteins specific to *B*. *cinerea*. Expressed sequence tag (EST) and proteomic analyses showed that 246 *S*. *sclerotiorum* secretome encoding genes had EST support, including 101 which were only expressed *in vitro* and 49 which were only expressed *in planta*, whilst 42 predicted proteins were experimentally proven to be secreted. These detailed *in silico* analyses of two important necrotrophic pathogens will permit informed choices to be made when candidate effector proteins are selected for function analyses *in planta*.

## Introduction

Phytopathogenic fungi form intimate associations with specific host plant species and cause disease. To be successful, each fungal pathogen, whether growing within or between living plant cells, or amongst dead plant tissue, must continually communicate with a susceptible host. Communication is achieved through the microbe secreting a cocktail of proteins, enzymes and metabolites which are either delivered into the plant cell or detected at the plant cell surface [1]. Secreted microbial proteins termed ‘effectors’ are produced during infection to; i) prevent host detection, ii) suppress the full activation of plant defences, iii) deal with the consequences of induced plant defences, or iv) induce plant cell death. For example, the small LysM domain containing proteins secreted by several fungal ascomycetes that interfere with chitin-induced plant immunity, promoting symptomless infection, such as Ecp6 from the biotrophic extracellular dwelling pathogen of tomato *Cladosporium fulvum* [2,3], Mg3LysM from the hemibiotrophic extracellular dwelling pathogen of wheat *Zymoseptoria tritici* [4] and Slp1 from the hemibiotrophic intracellular pathogen of rice *Magnaporthe oryzae* [5]. Only in a few instances are pathogenic fungi known to secrete effectors or metabolites to promote plant cell death, thereby enhancing disease formation. Two closely related cereal infecting necrotrophic fungal pathogens, *Stagnospora nodorum* and *Pyrenophora tritici-repentis*, which cause disease on wheat, secrete the ToxA host-selective proteinaceous toxin that induces host cell death and susceptibility in wheat genotypes that harbour the corresponding *Tsn1* toxin sensitivity gene [6,7]. Hence, it is often the communication events that occur at the initial stage of infection that have the most profound effect on the eventual outcome of the interaction [8].

*Sclerotinia sclerotiorum* is an economically important fungal pathogen that causes annual losses in excess of $200 million in the United States. *S*. *sclerotiorum* forms melanised hyphal aggregates called sclerotia, from which apotheica or fruiting bodies germinate and release pathogenic ascospores. *S*. *sclerotiorum* causes disease on over 400 plant species, including the crop species; oilseed rape (*Brassica napus)*, soybean (*Glycine max*), sunflower (*Helianthus annuus*) and many vegetable plants [9]. *S*. *sclerotiorum* is considered to cause disease on all hosts by using a solely necrotrophic lifestyle [10]. Typical symptoms of this fungal disease are observed from 3 days to up to 6 weeks post infection. Initially water soaked lesion occur at the initial site of infection, for example on the leaf of an oilseed rape plant. The lesions will then become necrotic and characteristic fluffy white mycelium will become visible. Wilting of the plant and tissue bleaching can be observed during the later stages of infection [11]. Central to the pathogenic strategy of *S*. *sclerotiorum* is the secretion of oxalic acid during infection. This seemingly simple organic acid regulates a range of functions related to infection including, lowering the pH to increase the activity of polygalaturonases [11], suppressing the plant oxidative burst [12] and altering the cellular redox status in the host plant [13]. By contrast, in *S*. *sclerotiorum* resistant crops, such as wheat (*Triticum aestivum*) and barley *(Hordeum vulgare*), there are elevated levels of germin or oxalate oxidase, an enzyme capable of breaking down oxalic acid into CO_2_ and H_2_O_2_ [14]. This lack of oxalic acid accumulation prevents infection progressing, due to the activation of a series of successful plant defences. Although the metabolite, oxalic acid, is a key player during this infection cycle, a few secreted proteins have also been identified to contribute to pathogenicity, namely two hydrolytic enzymes, SsCUTA (cutinase) [15] and Sspg1 (polygalacturonase) [15], a fungal integrin-like protein (SsITL) [16] and an uncharacterised hypothetical protein Ssv263 [17].

*Botrytis cinerea* is also an economically important plant necrotrophic pathogen of soft fruits, vegetables and flowers and is one of the most devastating plant pathogens of the grapevine. Its host range of over 200 plant species is comparatively smaller than *S*. *sclerotiorum* and disease symptoms are remarkably different. *B*. *cinerea* infection is characterised by grey masses of conidia which appear on infected fruit organs, including flowers, fruits, leaves, shoots and soil storage organs [18]. *B*. *cinerea* also produce small sclerotia in necrotic tissues from which conidiophores and multinucleate conidia form [18]. The growth of apothecia in the wild is very rare. The mechanisms permitting *B*. *cinerea* to infect dicotyledonous crop species is somewhat different from *S*. *sclerotiorum* and appears to involve the use of an arsenal of secreted cell wall degrading enzymes, the production of reactive oxygen species (ROS) via an NADPH oxidase [19], the secretion of two phytotoxins, namely botcinic acid and botrydial [20], a secreted cerato-platanin protein that induces plant cell death [21–23]. Also recently, Weiberg *et al*. [24] have demonstrated that *B*. *cinerea* can secrete small RNAs that suppress plant immunity by hijacking specific host RNAs.

A comparative analysis of the sequenced *S*. *sclerotiorum* and *B*. *cinerea* genomes revealed a high level of co-linearity and identity between these two closely related necrotrophs, in addition to a similar arsenal of genes associated with necrotrophic processes, such as plant cell wall degradation, in particular pectin, and oxalic acid production, plus the expansion *B*. *cinerea*-specific secondary metabolites [25]. In the original *S*. *sclerotiorum—B*. *cinerea* genome comparison, genes encoding for proteins with N-terminal signal peptides were identified by SignalP predictions, then the CAZyme and peptidase encoding genes were removed, resulting in 603 *S*. *sclerotiorum* and 879 *B*. *cinerea* genes encoding for candidate secreted effector proteins [25]. An additional recent *S*. *sclerotiorum* bioinformatics study, aimed at identifying effector-like proteins, combined evolutionary genetics and transcriptomics to predicted 486 *in planta* expressed secreted proteins and 70 *S*. *sclerotiorum*-specific candidate effectors [26]. However, this prediction was based on a limited interspecies comparison, while the secretome that was not expressed *in planta* was not explored, potentially excluding the involvement of the secretome in apothecia and/or sclerotia development. Subsequently, a complete, comparative, analysis of the entire *S*. *sclerotiorum* and *B*. *cinerea* secretome is required to characterise the repertoire, physical organisation, conservation and regulation of the secretomes from these two necrotrphic fungi.

Previously studies [27–29] combined several bioinformatics software to create a stringent pipeline which is able to predict the suite of secreted protein’s defined as a fungal secretome. The present study reports on the use of this pipeline to define and compare the entire *S*. *sclerotiorum* and *B*. *cinerea* secretomes. This comprehensive approach revealed the genomic organisation and functional profile of the secretomes, plus identified candidate effectors. A wide-ranging interspecies comparison of the *S*. *sclerotiorum* and *B*. *cinerea* secretomes with 115 fungi and oomycetes revealed the species specific and non-specific secreted proteins. Finally, *in vitro* and *in planta* expression patterns, plus proteomics analyses, provided support for the secretion of identified proteins and insights into possible function in infection and apothecia/ sclerotia development. By investigating the arsenal of proteins secreted by *S*. *sclerotiorum* and *B*. *cinerea*, a greater understanding of the necrotrophic infection process may be gained, while additional secreted proteins with biological relevance to fungal development and infection may be identified.

## Results

### The refined *S*. *sclerotiorum* and *B*. *cinerea* secretome

**Fig 1:**
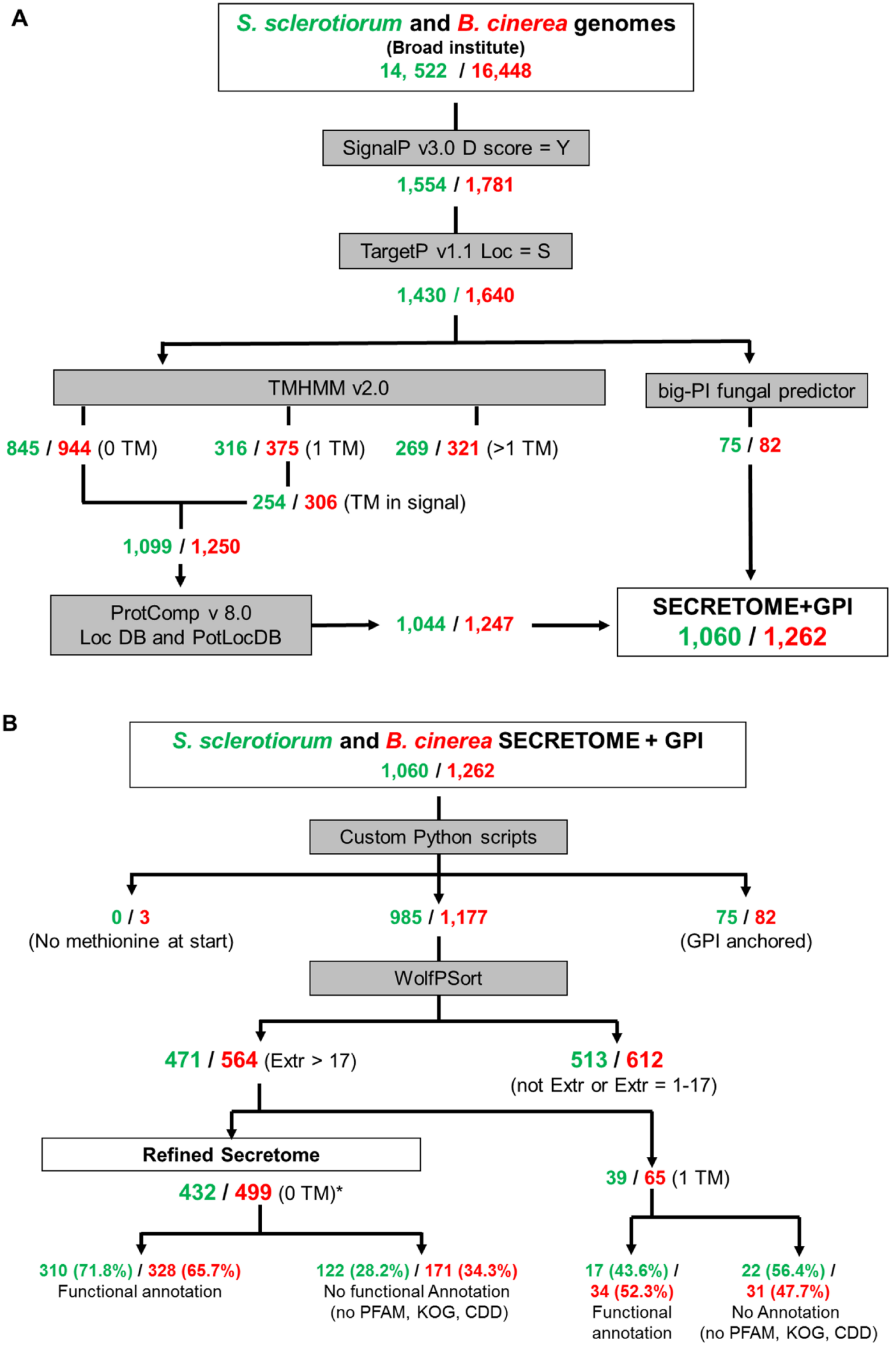
The bioinformatics pipeline used to predict the *S*. *sclerotiorum* and *B*. *cinerea* secretomes. (A) The total secretome. (B) The refined secretome. * denotes the removal of any mature proteins predicted to be shorter than 20 amino acids.

The *S*. *sclerotiorum* genome of the ‘1980’ strain (ATCC18683) and the *B cineria* B05.10 genome sequences were downloaded from the Broad Institute [25]. The present study initially used SignalP and TargetP in combination to predict the total secretome (Fig 1A), resulting in 1,430 and 1,640 secreted protein encoding genes, representing 9.8% and 10% of the respective *S*. *sclerotiorum* and *B*. *cinerea* genomes. Within this gene set, proteins predicted to contain no transmembrane domain (TM) or a single TM in, or just beyond, the signal peptides, plus GPI anchored proteins, were retained. Finally, a ProtComp analysis excluded proteins not predicted to be located in the extracellular space. This resulted in a predicted secretome size of 1,060 and 1,262 proteins for *S*. *sclerotiorum* and *B*. *cinerea* respectively (7.3% and 7.8% of total genome) (Table 1 in S1 File and Table 1 in S2 File). The second stage of the analysis identified the ‘refined secretomes’ using a more rigorous set of prediction tools (Fig 1B). All sequences that started with a methionine were utilised in a stringent WolF-PSort analysis, with an extracellular score >17, to increase the probability of such proteins being extracellularly secreted. This revealed refined *S*. *sclerotiorum* and *B*. *cinerea* secretomes of 432 and 499 protein encoding genes (Table 2 in S1 File and Table 2 in S2 File), representing approximately 3% of the two respective genomes.

### Physical distribution of the secretome-encoding genes across the *S*. *sclerotiorum* genome

**Fig 2:**
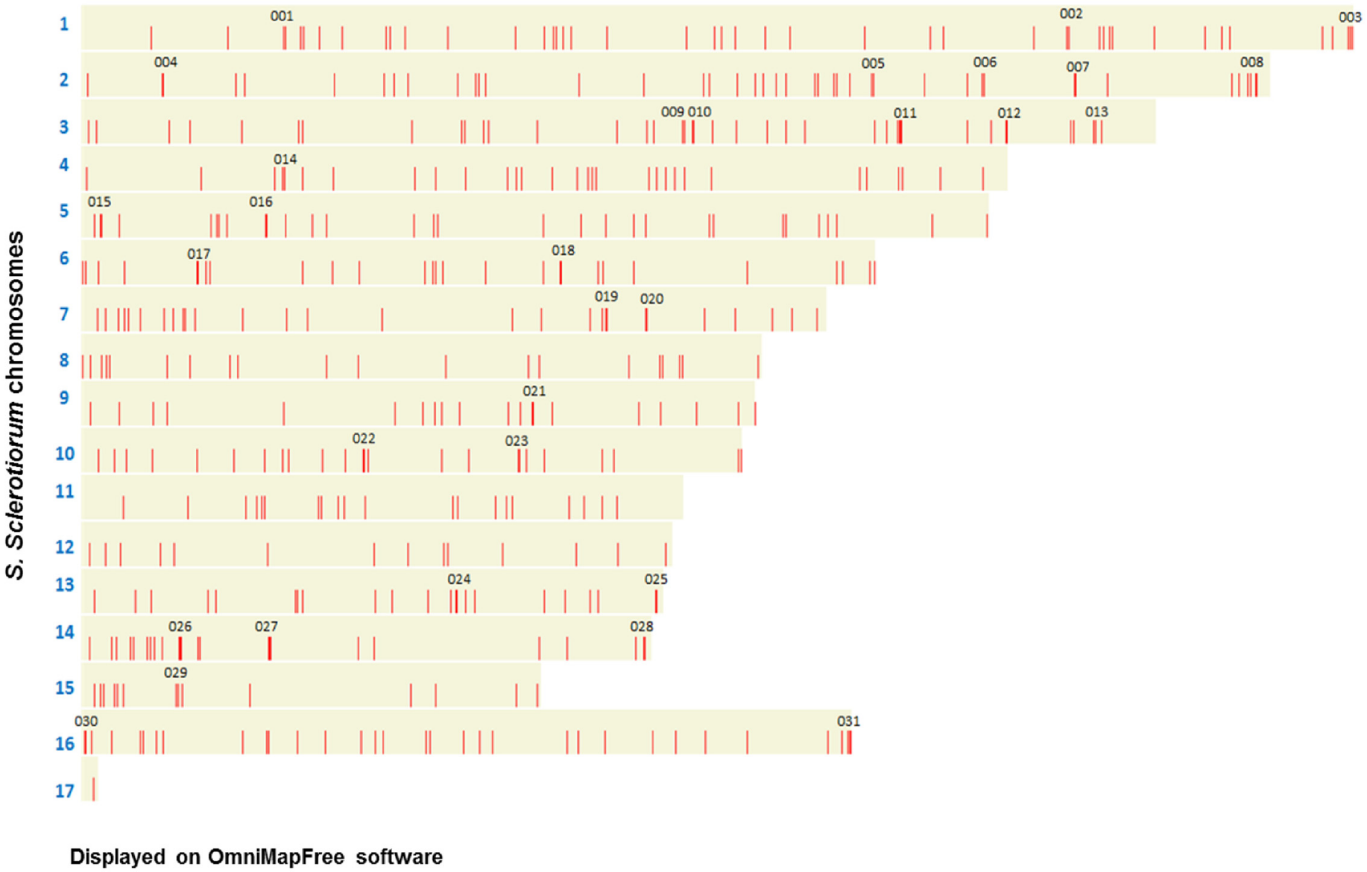
Distribution of the refined *S*. *sclerotiorum* secretome across the 16 mapped chromosomes and one pseudochromosome. Red bars show the genomic location of the refined *S*. *sclerotiorum* secretome-encoding genes. Numbers correspond to the 31 small gene clusters investigated for gene duplications and sequence relatedness.

The published *S*. *sclerotiorum* genome contains 16 linkage groups which correspond to an estimated 16 chromosomes [25], while a 17^th^ pseudochromosome containing the concatenated unmapped sequences was available. The *B*. *cinerea* genome consists of numerous contigs that remain to be physically mapped to chromosomes [25]. Therefore, the analysis of the physical distribution of the secretome-encoding genes across the respective chromosomes of the genome was only performed for *S*. *sclerotiorum*. The refined *S*. *sclerotiorum* secretome encoding genes were mapped onto the genome using OmniMapFree software (Table 1; Table 3 in S1 File). The entire refined secretome showed no obvious spatial pattern of distribution, with the genes appearing to be evenly spaced across all chromosomes (Fig 2). There was a single gene found within the pseudochromosome, SS1G_14515, a unique hypothetical protein with no functional annotation. Most chromosomes had a similar density of secretome genes, ranging from 10.66 to 13.76 genes per Mb. The overall density on chromosome 8 and 9 appeared to be slightly lower than on the other chromosomes, and for chromosome 12 the lowest density was observed.

**Table 1:** Distribution of the genes coding for predicted secreted proteins belonging to the refined secretome across the 16 chromosomes and 17^th^ pseudochromosome of *S*. *sclerotiorum*.

Chromosome Size (nt)		Genes			No. of secretome genes per Mb	Annotation of secreted proteins		
		Total predicted genes	Gene density per MB	Genes coding for secreted proteins		Yes	No	Unique to *S*. *sclerotiorum*
**1**	3964102	1479	373.1	44	11.10	27	17	1
**2**	3702977	1365	368.6	42	11.34	30	12	**4**
**3**	3347368	1278	381.8	40	11.95	33	7	0
**4**	2886255	1077	373.1	32	11.09	20	10	0
**5**	2826797	1040	367.9	30	10.61	23	7	0
**6**	2472283	908	367.3	25	10.11	17	10	1
**7**	2321737	876	377.3	27	11.63	20	7	0
**8**	2121402	800	377.1	20	**9.43** *	15	5	1
**9**	2098208	780	371.7	20	**9.53** *	15	5	1
**10**	2058163	773	375.6	24	11.66	17	6	0
**11**	1876643	696	370.9	20	10.66	14	6	1
**12**	1840947	684	371.5	14	**7.60** *	8	6	0
**13**	1812400	668	368.6	22	12.14	14	8	1
**14**	1774723	649	365.7	24	13.52	14	6	0
**15**	1431160	549	383.6	14	9.78	15	4	0
**16**	2398866	892	371.8	33	13.76	28	5	0
**17**	54754	8	146.1	1	**18.26** *	0	1	1
**Total**	38988785	14522	373.1	432		310	122	11

* Denotes those chromosomes which have above or below average number of secreted proteins per Mb.

The refined *S*. *sclerotiorum* secretome gene distribution pattern was inspected more closely to identify small physical gene clusters where two or more sequences were located directly next to each other or within a three gene proximity. In total, 31 small gene clusters were identified across the genome map (Table 2; Fig 2). Most of these predicted proteins were also of a considerable size > 200 amino acids. Interestingly, 5 gene clusters were located in close proximity to the ends of the chromosomes (clusters C003, C025, C028, C030 and C031) and a further two were located in the sub-telomeric regions (C008 and C015). A comparison with the composition of the functional clusters, as determined by Markov clustering (MCL) identified by Guyon *et al*. [26] showed five secretome genes (SS1G_01081, SS1G_11700, SS1G_05454, SS1G_00773, SS1G_00501), from four MCL clusters (MCL012, MCL047, MCL057 and MCL169), were located in five different physical gene clusters (C003, C009, C011, C019, C031) within the *S*. *sclerotiorum* genome. This showed that the genes encoding for the functionally related proteins identified in the MCL clusters by Guyon *et al*. [26] were predominantly physically separated on the *S*. *sclerotiorum* genome. None of the genes within a single cluster shared more than 45% identity, indicating that no cluster contained duplicated genes. Common PFAM domains were only found in two gene clusters. For example, SS1G_12499 and SS1G_12500 in gene cluster C017 both contained a serine carboxypeptidase domain (PF00450) which, while SS1G_00891 and SS1G_00892 in gene cluster C012 were both annotated as endoglucanases. Overall, this cluster analysis revealed that 16% of genes coding for the refined *S*. *sclerotiorum* secretome (i.e. 69 genes) resided in small, sequence heterogeneous, gene clusters that were distributed over 13 of the 16 predicted chromosomes.

**Table 2:** Description of the 31 gene clusters members from the *S*. *sclerotiorum* refined secretome.

Cluster	Gene_ID	Annotation
**C001**	SS1G_09841	PP
	SS1G_09844	PP
**C002**	SS1G_01426	PP
	SS1G_01428	pan domain containing protein
**C003**	SS1G_01081	catalase
	SS1G_01083	glycoside hydrolase family 31 protein
	SS1G_01086	PP
**C004**	SS1G_13035	PP
	SS1G_13036	multicopper oxidase
**C005**	SS1G_04662	alpha-galactosidase A precursor
	SS1G_04664	cell surface spherulin 4-like protein
**C006**	SS1G_04786	CHP
	SS1G_04790	acid phosphatase
**C007**	SS1G_12721	PP
	SS1G_12724	CHP
**C008**	SS1G_12930	glucan 1,3-beta-glucosidase precursor
	SS1G_12937	glycosyl hydrolase
	SS1G_12938	extracellular proline-serine rich protein
**C009**	SS1G_00501	endoglucanase A precursor
	SS1G_00505	CHP
**C010**	SS1G_00513	PP
	SS1G_00514	glycoside hydrolase family 26 protein
**C011**	SS1G_00768	PP
	SS1G_00772	HP similar to LysM domain-containing protein
	SS1G_00773	ankyrin repeat domain-containing protein 44
**C012**	SS1G_00891	HP similar to endoglucanase III
	SS1G_00892	exoglucanase-6A precursor
**C013**	SS1G_01003	PP
	SS1G_01005	alpha-glucosidase precursor
**C014**	SS1G_02345	PP
	SS1G_02347	alpha—glucanase mutanase
**C015**	SS1G_12057	polygalacturonase 1 precursor
	SS1G_12059	HP similar to endoglucanase B
**C016**	SS1G_12262	allergen Asp f 4 precursor
	SS1G_12263	carboxypeptidase
**C017**	SS1G_12499	Serine carboxypeptidase
	SS1G_12500	carboxypeptidase KEX1 precursor
**C018**	SS1G_07183	PP
	SS1G_07184	glycoside hydrolase family 32 protein
**C019**	SS1G_05449	carboxypeptidase cpdS precursor
	SS1G_05454	chitotriosidase-1 precursor
**C020**	SS1G_05493	HP similar to tannase and feruloyl esterase family protein
	SS1G_05494	wsc domain-containing protein
**C021**	SS1G_08644	lipase 5 precursor
	SS1G_08645	fad binding domain-containing protein
**C022**	SS1G_07655	subtilisin-like protein
	SS1G_07656	glycoside hydrolase family 61 protein
**C023**	SS1G_07836	HP similar to acidic protease
	SS1G_07837	PP
**C024**	SS1G_08889	glutaminase
	SS1G_08892	PP
	SS1G_08894	alpha beta-hydrolase
**C025**	SS1G_09129	6-phospho-beta-galactosidase
	SS1G_09130	CHP
**C026**	SS1G_09248	hydrophobin
	SS1G_09250	iron-sulfur cluster-binding rieske family domain protein
	SS1G_09251	HP similar to endoglucanase II
**C027**	SS1G_09363	-
	SS1G_09365	glucan 1,3-beta-glucosidase precursor
	SS1G_09366	periplasmic beta-glucosidase precursor
**C028**	SS1G_13385	actin patch protein 1
	SS1G_13386	cutinase
**C029**	SS1G_10165	carbohydrate esterase family 8 protein
	SS1G_10167	polygalacturonase 1
**C030**	SS1G_03610	CHP
	SS1G_03611	CFEM domain protein
**C031**	SS1G_11700	chitinase 1 precursor
	SS1G_11703	GPI transamidase component GPI16 precursor
	SS1G_11706	CHP

PP = predicted protein, HP = hypothetical protein, CHP = conserved hypothetical protein.

### Analysis of the attributes of the *S*. *sclerotiorum* and *B*. *cinerea* secretomes

**Fig 3:**
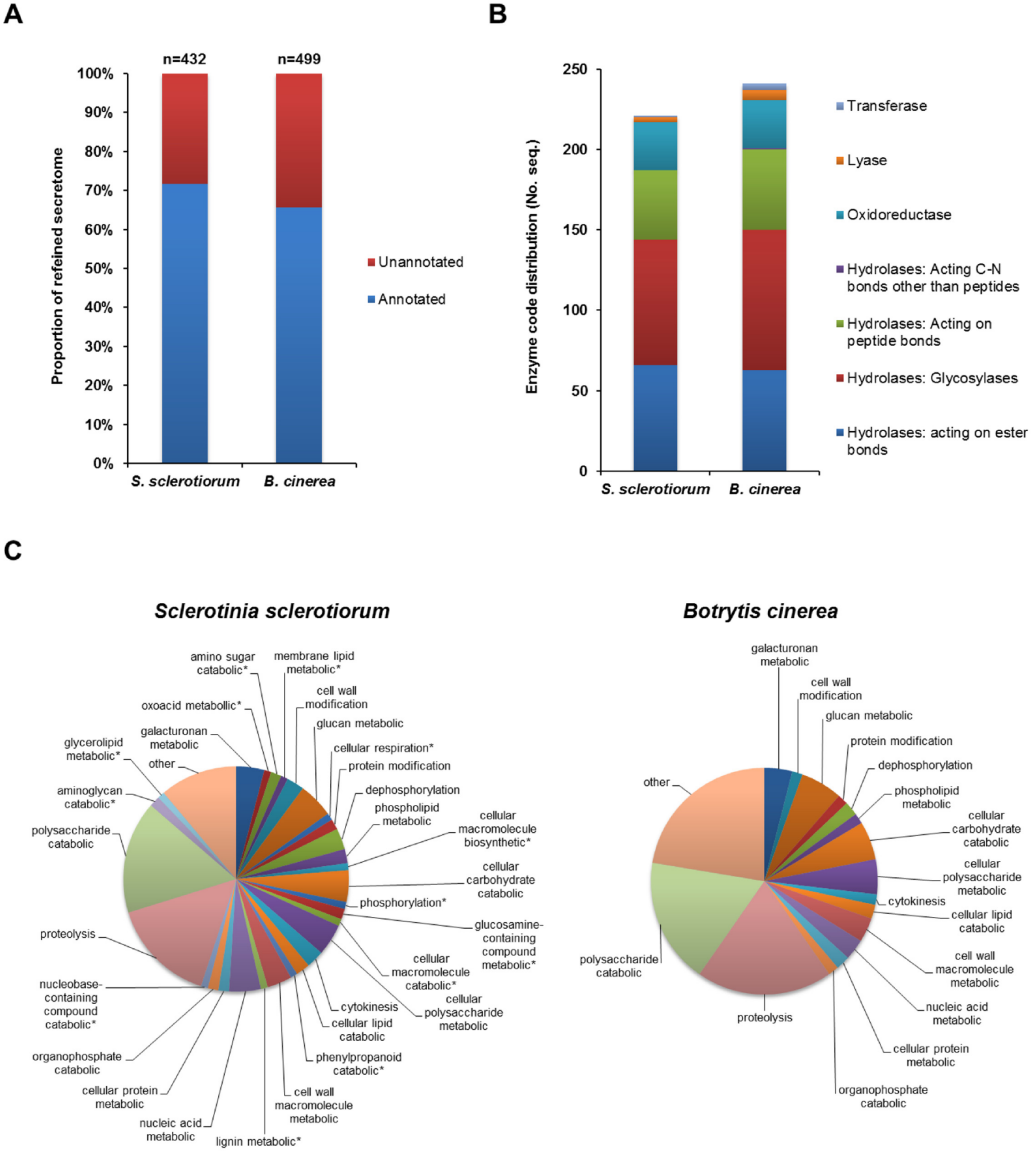
The profile of the *S*. *sclerotiorum* and *B*. *cinerea* secretomes. (A) The proportional representation of the annotated and unannotated secreted proteins in the refined secretomes. (B) The representation of major enzyme classes in the refined secretomes. (C) The representation of predicted biological processes (level 6 GO annotations) of the proteins within the refined *S*. *sclerotiorum* and *B*. *cinerea* secretomes. * denotes *S*. *sclerotiorum* specific GO annotations.

The refined secretome encoding genes from both species were analysed using Blast2GO, to retrieve updated annotations for the predicted proteins (e<-^5^). The Blast2GO analysis revealed that 310 *S*. *sclerotiorum* and 328 *B*. *cinerea* sequences had some form of annotation, i.e. an InterPro (IPO), Gene3D, SUPERFAMILY or PFAM entry, while 122 *S*. *sclerotiorum* and 171 *B*. *cinerea* sequences had no functional annotation. Proportionally, the *S*. *sclerotiorum* secretome possessed a slightly greater amount of functionally annotated proteins than *B*. *cinerea* (Fig 3A). Subsequent analyses separately focused on the annotated and non-annotated fractions of the respective secretomes.

The Blast2GO analysis revealed the general enzymatic activities (EC classification) and biological processes (GO annotations) of the refined *S*. *sclerotiorum* and *B*. *cinerea* secretomes (Table 4 in S1 File and Table 3 in S2 File). In both species the majority of sequences were involved in some form of catalytic activity primarily represented by hydrolyases, which targeted lipids, carbohydrates and proteins, and oxidoreductases (Fig 3B). The enzymes from the two secretomes were mapped onto the KEGG metabolic pathways (Table 3) revealing the high representation of enzymes involved in starch/ sucrose metabolism (for which *B*. *cinerea* showed a higher representation) and the pentose/ glucornate pathways, reflecting the abundance of enzymes required to breakdown plant polysaccharides. Evaluation of the biological processes assigned to the proteins within either secretome confirmed the high representation of polysaccharide catabolic and proteolytic processes in both secretomes, while also showing the greater diversification of function in the *S*. *sclerotiorum* secretome (Fig 3C).

**Table 3:** *S*. *sclerotiorum* secreted proteins predicted to be involved in KEGG metabolic pathways.

**KEGG pathway**	***S*. *sclerotiroum***		***B*. *cinerea***	
	Seq	Enz	Seq	Enz
Starch and sucrose metabolism	35	9	40	9
Pentose and glucuronate inter-conversions	22	4	23	3
Drug metabolism	14	1	14	1
Other glycan degradation	9	5	9	5
Glycine, serine and threonine metabolism	8	1	8	1
Phenylpropanoid biosynthesis	7	2	11	2
Aminobenzoate degradation	6	2	4	2
Riboflavin metabolism	6	1	4	1
Phenylalanine metabolism	5	1	7	1
Sphingolipid metabolism	5	3	7	4
Amino sugar and nucleotide metabolism	5	3	6	3
Galactose metabolism	4	2	5	2
Glycosaminoglycan degradation	4	2	4	2
Glycerolipid metabolism	4	2	6	2
Glycosphingolipid biosynthesis—ganglio series	4	2	4	2
Fructose and mannose metabolism	2	1	2	1
Biosynthesis of antibiotics	2	2	-	-
Various types of N-glycan biosynthesis	2	2	2	2
Cyanoamino acid metabolism	2	1	6	3
Glycosphingolipid biosynthesis—globo series	2	2	3	2
Glyoxylate and dicarboxylate metabolism	2	2	1	1
TCA cycle	1	1	-	-
Ether lipid metabolism	1	1	2	1
Pentose phosphate pathway	1	2	-	-
Methane metabolism	1	1	-	-
N-glycan biosynthesis	1	1	1	1
Tryptophan metabolism	1	1	-	-
Inositol phosphate metabolism	-	-	1	1
Alanine, aspartate and glutamate metabolism	-	-	1	1
Steroid hormone biosynthesis	-	-	1	1
Glutathione metabolism	-	-	1	1
Phosphatidylinositol signalling system	-	-	1	1
Fatty acid elongation	-	-	1	1
Taurine and hypotaurine metabolism	-	-	1	1

Seq = number of fungal sequences, Enz = number of enzyme classes

#### Identification of known *S*. *sclerotiorum* and *B*. *cinerea* secreted virulence factors

To validate the refined secretome predictions, secreted proteins known to be required for full virulence in *S*. *sclerotiorum* and *B*. *cinerea* were identified. Four *S*. *sclerotiorum* proteins required for infection were identified in the refined secretome, including the SsCUTA cutinase, the Sspg1 polygalacturonase, a fungal integrin-like protein SsITL, and a novel hypothetical protein, Ssv263 (Table 4). Additionally, four LysM domain containing proteins were identified in the *S*. *sclerotiorum* genome, of which three were identified in the refined secretome. The excluded LysM containing protein, SS1G_03535, was identified in the total secretome but not in the refined secretome due to an extracellular Wolf-PSort score of 12, which is below the threshold set at 18. This protein has been described to be similar to the CfECP6 effector protein [2]. There are no studies on the function of these four LysM proteins identified in the *S*. *sclerotiorum* secretome. However, one LysM containing protein, SS1G_00772, resided in the small gene cluster C011 on chromosome 3, while the other two, SS1G_12509 and SS1G_12513, were located closely together on chromosome 6. Interesting SS1G_12509 resided next to a chitinase and may therefore be important as LysM domain containing effector proteins are involved in suppressing chitin-mediated plant defences.

**Table 4:** The identification of *S*. *sclerotiorum* and *B*. *cinerea* secreted proteins confirmed to be required for full virulence on plant host.

Broad ID	Gene name	Function	Virulence decreased	Refined secretome	Ref.
SS1G_00263	ssv263	Hypothetical protein unique to *S*.*sclerotiorum* and *B*.*cinerea*	Y	Y	[17]
SS1G_07661	SsCUTA	Cutinase enzyme	Y	Y	[15]
SS1G_10167	sspg1	Polygalacturonase	Y	Y	[15]
SS1G_14133	SSITL	Fungal integrin-like protein	Y	Y	[16]
SS1G_02462	abx	Arabinofuranosidase/beta-xylosidase	Y	N	[73]
SS1G_03535	LysM domain	Contains a LysM domain (e = 0.024) (similar to ECP6- but WolfPsort = ext12 in total secretome)	-	N	Reported to be similar to ECP6 [2]
SS1G_00772*	LysM domain	Contains a LysM domain (e = 0.0028)	-	Y	Reported to be similar to ECP6 [2]
SS1G_12509	LysM domain	Contains a LysM domain (e = 0.12)	-	Y	Reported to be similar to ECP6 [2]
SS1G_12513	LysM domain	Contains a LysM domain (e = 0.03)	-	Y	Reported to be similar to ECP6 [2]
BC1G_02163	Bcspl1	Phytotoxic cerato-platanin, elicitor of host cell hypersensitive response	Y	Y	[23]
BC1G_03590	Xyn11A	Endo-beta-1,4-xylanase	Y	Y	[74]
BC1G_11143	Bcpg1	Endopolygalacturonase	Y	Y	[75,76]
BC1G_00230	Bcpg2	Endopolygalacturonase	Y	Y	[75,76]
BC1G_00617	Bcpme1	Pectin methyl esterase	Y	Y	[77]

* SS1G_00772 identified in cluster C011

Within the *B*. *cinerea* secretome five known virulence factors were identified including; the Bcspl1 cerato-platanin, the Xyn11A endoxylanase, two polygalacturonases, Bcpg1 and Bcpg2, plus the pectin methylesterase Bcpme1 (Table 4). The *B*. *cinerea* cerato-platanin is required for full virulence and has been shown be to cause necrosis and chlorosis on the plant leave [21–23]. Interestingly, an uncharacterised cerato-platanin (PF07249) domain containing gene (SSG1_10096) was also identified in the *S*. *sclerotiorum* secretome. In addition, cerato-platanins have been shown to be required for full virulence in plant pathogens *M*. *oryzae* [30] and *Colletotrichum hinginsianum* [31] and mycoparasite *Hypocrea atroviridis* [32].

#### Comparative PFAM analyses of *S*. *sclerotiorum* and *B*. *cinerea*

A comparison of the most abundant PFAM domains in the two species revealed potential functional similarities and differences in the refined secretomes (Tables 5 and 6). Out of the 432 *S*. *sclerotiorum* and 499 *B*. *cinerea* secretome protein sequences, 289 and 302 contained at least 1 PFAM domain (Table 2 in S1 File and Table 2 in S2 File). The most abundant PFAM domains included hydrolytic enzymes involved in the breakdown of host plant cell walls. In the *S*. *sclerotiorum* and *B*. *cinerea* secretomes, 25 and 31 glycoside hydrolase families were identified among 90 and 101 genes, representing 21% and 20% of the refined secretomes (Table 5 in S1 File and Table 4 in S2 File). In *S*. *sclerotiorum* these hydrolytic enzyme encoding genes were evenly distributed across the genome. One of the most abundant PFAM domains in both the refined secretomes was the GH28 (PF00295) (Table 5), which was found in 17 proteins predicted to have polygalacturonase activity including the characterised pathogenicity factor (Sspg1; Bcpg1/2) [15,33]. A carbohydrate-binding module that associates with glycoside hydrolases (PF00734) was expanded in the *S*. *sclerotiorum* secretome, which had 17 domains in comparison to the six found in *B*. *cinerea*. In contrast, the number of tannase/ feruloyl esterase domains (PF07519) was increased in the *B*. *cinerea* secretome compared to *S*. *sclerotiorum*. The refined *S*. *sclerotiorum* and *B*. *cinerea* secretomes also respectively contained 24 and 33 genes involved in lipid degradation and 37 and 32 genes involved in protein degradation (Table 5 in S1 File S1 and Table 4 in S2 File) similar to the *F*. *graminearum* secretome [28]. The most abundant PFAM domain, in this set of sequences, was a type B carboxylesterase (PF00135) implicated in lipid degradation (Table 5). This domain was even more abundant in the *B*. *cinerea* secretome with 18 domains compared to the eight identified in the *S*. *sclerotiorum* secretome. The abundance of peptidases was predominantly conserved between species, while PF00082 was expanded in *S*. *sclerotiorum* (Table 5).

**Table 5:** The most common PFAM domains predicted to be involved in degradation of host plant substrate found in both the *S*. *sclerotiorum* and *B*. *cinerea* refined secretomes.

**PFAM definition**	**PFAM**	***S*. *sclerotiorum***	***B*. *cinerea***
Glycoside hydrolase, family 28	PF00295	17	17
Cellulose-binding domain, fungal	PF00734	17	6
Glycoside hydrolase, family 5	PF00150	9	6
Carboxylesterase, type B	PF00135	8	18
Peptidase S53, propeptide	PF09286	8	8
Peptidase S8/S53 domain	PF00082	7	2
Glycoside hydrolase, family 61	PF03443	7	7
Peptidase A1	PF00026	6	7
Peptidase S10, serine carboxypeptidase	PF00450	6	7
Lipase, GDSL	PF00657	6	2
Glycoside hydrolase family 3 C-terminal domain	PF01915	5	9
Glycoside hydrolase, family 45	PF02015	5	1
Pectate lyase/Amb allergen	PF00544	4	5
Glycoside hydrolase, family 18, catalytic domain	PF00704	4	3
Cutinase	PF01083	4	7
Pectinesterase, catalytic	PF01095	4	3
Glycoside hydrolase, family 71	PF03659	4	4
Glycoside hydrolase, family 76	PF03663	4	2
Glycosyl hydrolase, family 13, catalytic domain	PF00128	3	3
Glycoside hydrolase, family 11	PF00457	3	2
Tannase/feruloyl esterase	PF07519	2	8
Alpha/beta hydrolase fold	PF07859	0	6

**Table 6:** The most abundant PFAM domains within the *S*. *sclerotiorum* and *B*. *cinerea* secretome that do not have plant cell hydrolytic properties.

**PFAM definition**	**PFAM**	***S*. *sclerotiorum***	***B*. *cinerea***
Glucose-methanol-choline oxidoreductase	PF00732; PF05199	10	12
Histidine phosphatase superfamily, clade-2	PF00328	6	4
FAD linked oxidase, N-terminal	PF01565	6	7
Multicopper oxidase	PF00394; PF07731; PF07732	5	3
Carbohydrate-binding WSC	PF01822	5	3
Fibronectin type III-like domain	PF14310	5	8
Chitin-binding, type 1	PF00187	4	1
Extracellular membrane protein, CFEM domain	PF05730	4	0
Tyrosinase	PF00264	3	3
Chloroperoxidase	PF01328	3	5
Berberine/berberine-like	PF08031	3	4
Cupin 1 and 2	PF00190	3	4
PAN-1 domain	PF00024	2	0
Metallophosphoesterase domain	PF00149	2	0
CAP domain	PF00188	2	2
Ribonuclease T2-like	PF00445	2	2
S1/P1 nuclease	PF02265	2	1
Necrosis-inducing protein	PF05630	2	2
Hydrophobic surface binding protein A	PF12296	2	3
Ferritin-like domain	PF13668	2	3
S1/P1 Nuclease	PF02265	2	1
Cerato-ulmin hydrophobin family	PF06766	1	1
Cerato-platanin	PF07249	1	2
GLEYA adhesin domain	PF10528	1	1
Haem peroxidase	PF00141	1	2
Hydrophobin	PF06766	1	1

Beyond the domains potentially involved in plant cell degradation, the Blast2GO analysis describes numerous proteins involved oxidation-reduction processes within the *S*. *sclerotiorum* and *B*. *cinerea* secretomes (Tables 4 and 5 in S1 File S1 and Table 3 and 4 in S2 File). Oxidoreductases are a large protein family which catalyse the transfer of electrons between molecules and are involved in a range of processes [34]. A glucose-methanol-choline (GMC) oxidoreductase domain (PF00732) was highly present (12 proteins) in the *B*. *cinerea* secretome, which was comparable to the 10 protein in the *S*. *sclerotiorum* secretome. Multiple FAD linked oxidase (PF01565) domains were identified within the *S*. *sclerotiorum* and *B*. *cinerea* secretomes. These enzymes use FAD as a co-factor and are mainly oxygen-dependent oxidoreductases (IPR006094). In fungi, these enzymes are involved in many process including the formation and stability of spores, in defence and virulence mechanisms, and in browning and pigmentation, mainly melanin production [35,36]. Several multi-copper oxidases were identified which have laccase activity and are involved in the oxidation of phenolic lignin units, while these proteins many also have other function during fungal development, melanin synthesis and detoxification, plus plant pathogenesis [37]. Several chloroperoxidases (PF01328) were identified in the *S*. *sclerotiorum* and *B*. *cinerea* secretomes. This protein is a heme-containing glycoprotein that is secreted by various fungi (IPR000028) and performs a range of diverse functions including facilitating the decomposition of hydrogen peroxide to oxygen and water and catalysing chloroperoxidase P450-like oxygen insertion reactions (IPR000028). These enzymes have also been described to have lignin degradation activity as they are potential chlorinators of lignin [38,39].

Additional pathogenicity-related PFAM domains were identified in the two refined secretomes. Two cupin domains (PF00190) were present in the oxalate decarboxylase enzymes in the *S*. *sclerotiorum* and *B*. *cinerea* secretomes, which are crucial for the degradation of oxalic acid, which is secreted at very high levels and could become potentially toxic if the pathogen does not locally hydrolyse this pathogenicity determinant [40,41]. In addition, in *S*. *sclerotiorum* the degradation of oxalic acid is important for the regulation of pH-dependent expression of genes during a later stage of the infection process [11,42]. Both the *S*. *sclerotiorum* and *B*. *cinerea* secretomes included the necrosis-inducing protein (PF05630) plus the cerato-ulmin hydrophobin (PF06766) and the cerato-platanin (PF07249). Interestingly, the *S*. *sclerotiorum* secretome possessed more chitin-binding domains (PF00187) than *B*. *cinerea*, which also lacked any LysM (PF01476), CFEM (PF05730), PAN-1 (PF00024) or metallophosphoesterase (PF00149) domains. Hence, the *S*. *sclerotiorum* secretome has an increased representation of several effector-like protein domains.

#### Identification of effector motifs

Effectors of plant infecting oomycetes and animal infecting malaria parasites, but not fungi, possess a conserved RXLR-dEER motif that facilitates secretion and uptake into the host cell, resulting in the modification of host transcription [43–46]. A strong candidate for convergent evolution amongst intracellular non-necrotrophic fungi is the degenerate Y/F/WxC motif, which is abundant within the secretomes of the barley leaf infecting ascomycete *Blumeria graminis* f. sp. *hordei* and wheat infecting basidiomycete rust species, *Puccinia graminis* f. sp. *tritici* [47]. Formal proof of a role in virulence for secreted proteins containing a Y/F/WxC motif in any species is eagerly anticipated [48].

The refined *S*. *sclerotiorum* and *B*. *cinerea* secretomes were inspected for protein sequences containing RXLR, dEER and Y/F/WxC motifs and their proximity to the N-terminal signal peptide. In total, 21 *S*. *sclerotiorum* and 18 *B*. *cinerea* proteins from the refined secretomes contain a RXLR motif (Table 6 in S1 File and Table 5 in S2 File). Four *S*. *sclerotiorum* and six *B*. *cinerea* sequences contained a RXLR motif within 55 base pairs of the signal peptide. In both species this included predicted proteolytic enzymes (SS1G_00624, BC1G_07149, BC1G_12343), dioxygenases (SS1G_03653, BC1G_05479) and unannotated proteins (SS1G_14321, BC1G_03560, BC1G_12732). These RXLR containing sequences did not possess a dEER motif downstream of the RXLR domain. None of these *S*. *sclerotiorum* genes were found in the physical gene clusters.

Eight *S*. *sclerotiorum* and 11 *B*. *cinerea* protein sequences contained a Y/F/WxC motif within 16 and 20 base pairs of the signal peptide, respectively (Table 7 in S1 File and Table 6 in S2 File). In *S*. *sclerotiorum* these mature proteins ranged from 232 to 1020 amino acids in length and each sequence contained 6, or more, cysteine residues. Six of the sequences were annotated and consisted of different enzymes including, glycoside hydrolases (GH45), an aspartate protease and a histidine acid phosphatase. Of these, SS1G_04662, an alpha-galactosidase was found in gene cluster C005 (Fig 3; Table 3). In *B*. *cinerea* these proteins ranged between 41 and 912 amino acids in length, included four small unannotated cysteine-rich proteins, four enzymes consisting of a glycoside hydrolases (GH45), a carboxylesterase, an aminopeptidase and a TOS1-like glycosyl hydrolase, plus a carbohydrate binding module. In both species, a homologue of candidate effector 5, from the apple scab fungus *Venturia inaequalis* [49] and the grapevine dieback fungus *Eutypa lata* [50], with a Y/F/WxC motif.

#### Analysis of the unannotated S. sclerotiorum and B. cinerea secretomes

No recognised PFAM domains or IPS entries were identified in the 122 and 171 unannotated *S*. *sclerotiorum* and *B*. *cinerea* secretomes. Both sets of unannotated sequences were inspected for the presence of small (less than 200 amino acids), cysteine-rich proteins (5% or greater), a sequence profile that has previously been used to predict candidate effector genes [3,29]. Effectors previously discovered which fit this sequences profile include CfECP6, an effector which binds chitin during *C*. *fulvum* plant infection and prevents PAMP triggered recognition by the plant chitin receptors [2,3]. Three putative homologues of CfECP6 were recently functionally characterised in the wheat infecting fungus *Z*. *tritici* [4]. In the *S*. *sclerotiorum* refined secretome, 38 proteins had >5% cysteine residues of which 22 were less than 200 amino acids in length and had 6 or more cysteine residues in the mature protein (Table 8 in S1 File). None of these small cysteine-rich proteins were found to contain either an RXLR or Y/F/WxC motif. Eighteen of these proteins had no annotation. When all 22 *S*. *sclerotiorum* cysteine-rich proteins were mapped to the genome, no genes were present on chromosomes 10, 11, 12, 13 and 15, which is a similar distribution pattern to that of the RXLR and Y/F/WxC motif containing proteins. The genes coding for the cysteine-rich proteins SS1G_01003, SS1G_02345, SS1G_03611 and SS1G_09248 were found in gene clusters C013, C014, C030 and C026, respectively. Within the 171 *B*. *cinerea* unannotated proteins, 26 proteins were predicted to possess > 5% cysteine residues in a mature amino acid sequence of less than 200 amino acids, three of which possessed a Y/F/WxC motif (Tables 6 and 7 in S2 File).

### Multiple species comparative analyses

A multispecies comparison was done to explore the relatedness of the refined *S*. *sclerotiorum* and *B*. *cinerea* secretomes to the predicted proteomes of 115 other species including fungal and oomycete plant or animal pathogens, plus some free living eukaryotic organisms (Tables 1 and 2 in S3 File). The genomes of 115 species were compared to the refined secretomes for *S*. *sclerotiorum* and *B*. *cinerea* via BlastP using e^-5^ and e^-100^ similarity values to identify secreted protein encoding genes with a high level of sequence similarity across a wide taxonomical distribution.

#### Identification of the secreted proteins specific to S. sclerotiorum and B. cinerea

Eleven protein sequences were found to be specific to the *S*. *sclerotiorum* secretome (Table 3 in S3 File) and 32 protein sequences were found to be unique to the *B*. *cinerea* secretome (Table 4 in S3 File S3). All genes had only single copies in the respective genomes, except for BC1G_12747 which had two copies. There was no significant enrichment of PFAM domains in any of these proteins sequences, while SS1G_13126 was a small cysteine-rich protein. Three of the unique *S*. *sclerotiorum* proteins had EST support. None of these *S*. *sclerotiorum* specific genes were found in the 31 gene cluster and were evenly distributed across 8 chromosomes, with 4 residing on chromosome 2. Six of the 32 *B*. *cinerea* specific genes were small, cysteine-rich, proteins.

#### Identification of the S. sclerotiorum and B. cinerea shared secretome

The entire multispecies dataset was explored for shared *S*. *sclerotiorum* and *B*. *cinerea* proteins not found in the other species using a threshold of e^-5^. This revealed 30 proteins uniquely shared by these two species (Table 5 in S3 File). To further explore the 30 *S*. *sclerotiorum* proteins potentially shared with *B*. *cinerea*, a BlastN search was done using the 30 *S*. *sclerotiorum* nucleotide sequences to find the homologous gene in the *B*. *cinerea* genome. The predicted amino acid sequences for the two IDs were then aligned to ensure that these were gene homologues. Six proteins had maximum identity values above 40% and 24 genes had a maximum identity value above 50% (Table 6 in S3 File). This represents a high confidence level of homology between the two gene sequences. Four of these *S*. *sclerotiorum/ B*. *cinerea* proteins were small, cysteine-rich proteins identified earlier (SS1G_02068/BC1G_02834, SS1G_03897/BC1G_04521, SS1G_09175/BC1G_01059 and SS1G_12648/BC1G_04660). Most genes had single copies within the genomes except for SS1G_00263/BC1G_00896, SS1G_01086/BC1G_12867, and SS1G_04312/BC1G_12229 where 2 copies are found in both species. SS1G_09841, SS1G_01086 and SS1G_00768 were found in the identified gene clusters C001, C003 and C011, respectively.

#### Interspecies secretome comparison

The refined *S*. *sclerotiorum* and *B*. *cinerea* secretomes contain at least one gene homologue (e^-100^) in 110 and 111 of the 115 species, respectively (Table 1 in S3 File). When the refined *S*. *sclerotiorum* secretome was compared to all other genomes at a lower level of stringency (e^-5^) (Table 1 in S3 File), *B*. *cinerea* shared the highest number of common sequences (92%). This result was anticipated because these two species share 84% of their total proteomes [25]. The next most similar proteome to the refined *S*. *sclerotiorum* secretome was *Botryosphaeria dothidea*, an ascomycete plant pathogen which infects mainly woody species and shrubs. This proteome shares approximately 77% homology (331 proteins) with the *S*. *sclerotiorum* secretome (Table 1 in S3 File). Many ascomycete plant pathogens which infect different monocotyledonous and dicotyledonous plants also shared a high level of similarity with the *S*. *sclerotiorum* secretome (Table 1 in S3 File). The ascomycete plant pathogens, share between 42% and 76% of their proteome’s with the *S*. *sclerotiorum* secretome. Curiously, a single plant saprophyte, *Hysterium pulicare*, a dothideomycete found to live in decaying woody material shared 319 proteins (73%) with *S*. *sclerotiorum*. The citrus infecting pathogen, *Rhytidhysteron rufulum*, which is also a dothideomycete shared 308 proteins (71%) with *S*. *sclerotiorum*. Monocotyledonous infecting ascomycete pathogens such as *Fusarium verticillioides*, *Pyrenophora tritici-repentis* and *Gaeumannomyces graminis* shared 596 (56%), 551 (52%) and 496 (47%) proteins respectively.

The refined *S*. *sclerotiorum* and *B*. *cinerea* secretomes shared approximately 67% of sequences with Aspergilli and 75% with *Fusarium oxysporum*, all of which have a saprophytic plus either an animal pathogenic or plant pathogenic lifestyles that is very different to *S*. *sclerotiorum* [51,52]. Although Aspergilli and *F*. *oxysporum* can produce oxalic acid in large quantities *in vitro* [40], similarly to *S*. *sclerotiorum* and *B*. *cinerea*, the role of this secreted metabolite in animal and plant pathogenicity is unclear [53]. There was no relationship between the homology of the proteomes between other oxalic acid producing fungi and the refined *S*. *sclerotiorum* and *B*. *cinerea* secretomes. Other phyla of fungi including basidiomycetes share much less homology with the *S*. *sclerotiorum* (less than 60%). The chromalveolata phyla which contain oomycete plant pathogens such as *Phytophthora spp* share no greater than 36% proteome homology with the *S*. *sclerotiorum* secretome.

A comparison between the refined secretome of *S*. *sclerotiorum* and the proteome’s of *Alternaria brassicicola* and *Leptosphaeria maculans* was made to explore putative secreted proteins uniquely shared by these three fungal species which are all economically important pathogens of oilseed rape. No proteins unique to only these three species were found. A second specific investigation was made to explore plant pathogens that infect multi dicotyledonous and monocotyledonous species. Again, no genes were specific to this entire group. Although three genes found in the *S*. *sclerotiorum* and *B*. *cinerea* inter-comparison were subsequently detected only in a limited number of other fungi (Table 7).

**Table 7:** Genes found in both *S*. *sclerotiorum* and *B*. *cinerea* refined secretomes that are also present in only a limited number of other fungi. Blast value; e^-5^.

Gene	Function	Species	Lifestyle	Host
**SS1G_00849**/BC1G_09054	Conserved hypothetical protein	*Chaetomium globosum*	Sord-sap/animal	Woody/soil
		*Colletotrichum graminicola*	Sord-plant path	Dicot
		*Colletotrichum higginsianum*	Sord-plant path	Dicot
		*Magnaporthe oryzae*	Sord-plant path	Monocot
		*Magnaporthe poae*	Sord-plant path	Monocot
		*Fusarium spp*	Sord-plant path	Mono/Dicot
**SS1G_09196/**BC1G_11545	Hypothetical protein similar to enoyl- hydratase isomerase	*Colletotrichum higginsianum*	Sord-plant path	Dicot
		*Fusarium graminearum*	Sord-plant path	Mono/Dicot
**SS1G_12262/** BC1G_01872	Allergen Asp f 4 precursor	*Blastomyces dermatitidi*	Saprophyte/ animal pathogen	
		*Histoplasma capsulatum*		
		*Paracoccidioides brasiliensis*		
		*Penicillium chrysogenu*		

### Transcriptional and proteomic support for the *S*. *sclerotiorum* secretome

In order to provide transcriptional support for the refined *S*. *sclerotiorum* secretome seven EST libraries and a microarray analysis which compared sunflower cotyledon infection with *in vitro* growth were inspected (Table 9 in S1 File). Out of the 432 genes in the refined secretome, 58 genes had at least one hit in the developing sclerotia library (G781), 66 genes had hits in the mycelium library (G786), 95 genes in the developing apothecia library (G787), 20 genes in the infected *Brassica* library (G865), 21 genes in the infection cushion library (G866), 146 genes in the infected tomato library (G2118) and 118 genes in the oxidative stress library (G2128). Collectively, this EST analysis revealed that 251 genes present in the refined secretome (58%) had EST support, and of these 101 where found only to be expressed *in vitro*, including during apothecia and/or sclerotia development, and 49 were found to be only expressed *in planta*. Similar to the EST analysis, 252 (59%) genes in the secretome were detected in the microarray analysis. Only 72 (17%) secretome-encoding genes showed a significant modulation in transcription post sunflower infection, including 44 up-regulated genes which predominantly encoded hydrolytic enzymes, and 28 down-regulated genes which mainly encoded hypothetical protein.

Additionally, the refined *S*. *sclerotiorum* secretome was compared to two proteomic studies which used ESI-q-TOF MS/MS and LC–MS/MS to identify secreted proteins in liquid cultures [54] or exudates found in the sclerotial liquid which encases immature sclerotia [55]. Out of the 14 proteins identified in the liquid culture, 10 proteins were identified in the refined secretome (71%) (Table 10 in S1 File). The later proteomics study of the sclerotial liquid identified 56 proteins, 32 of these were sequences predicted in the refined secretome (57%) (Table 11 in S1 File). The refined *S*. *sclerotiorum* secretome so far contains 42 proteins that have been experimentally confirmed to be secreted.

### Infection and developmentally regulated *S*. *sclerotiorum* secreted proteins

*S*. *sclerotiorum* sequences with hits above 40 in any of the EST libraries were investigated further to determine which genes were the most expressed under the seven different conditions explored. Twenty seven genes were identified with 40 or more hits in at least one EST library. Fifteen of the sequences with this high level of EST support have PFAM annotation and 12 sequences had no PFAM domains or IPS entries (Table 12 in S1 File).

Twenty GHs encoding genes had good EST support in both the infected Brassica library (G865) and the tomato infection library (G2118), compared to 11 genes which had only low EST support in the neutral pH mycelia library (G786). This result highlights the possible specific role of these 20 proteins in plant infection rather than just fungal growth. Interestingly, SS1G_03611 only had EST support in the plant infection libraries (G865, G2118) and the infection cushion library (G866). SS1G_03611 was predicted to encode a mature protein of 101 amino acids in length with a cysteine residue content of 7.93%. This gene was found in the small gene cluster C030 (Fig 3) and was predicted to possess a CFEM domain which is a specific cysteine-rich domain found in some proteins with proposed roles in fungal pathogenesis or conserved fungal effector domains [56]. The experimentally proven secreted virulence factors Sspg1 and Ssv263 had high EST counts of 76 and 61 specifically during tomato infection, while SsCUTA and the predicted LsyM-containing proteins had very weak EST support in all libraries (Tables 9 and 12 in S1 File). Seventeen RXLR or Y/F/WxC motif-containing proteins had EST support, while the RXLR containing gene, SS1G_04725, had an EST count > 40 specifically during apothecia development and two Y/F/WxC containing genes, SS1G_03268 and SS1G_13860, has EST support > 10 during sclerotia development and oxidative stress, respectively (Tables 9 and 12 in S1 File). The CAP (cysteine-rich secretory proteins, antigen 5 and pathogenesis-related 1) family protein encoded by SS1G_03326 had 97 EST hits in the sclerotia development library (G781). This protein family has been shown to have many roles in regulation of extracellular matrix, branching morphogenesis and cell wall loosening, potentially as either a proteases or protease inhibitors which have possible antifungal activity [57]. All of these described physiological processes would happen during sclerotia formation. Similarly, SS1G_04725 which is predicted to be involved in the extracellular synthesis of browning pigments, such as melanin, had 46 EST counts in the developing apothecia library (G787). Of the protein with no functional annotation, SS1G_06412 which is unique to *S*. *sclerotiorum* only had EST support in the apothecia development library (G787), while SS1G_13599 had an extremely high EST count of 675 in the developing sclerotia library G781, suggesting that these proteins have roles in these developmental processes.

Of the 69 genes coding for members of the refined *S*. *sclerotiorum* secretome that are predicted to reside within small gene clusters, 8 have high EST support > 40 counts and 7 genes are supported by between 10 and 39 counts and a further 24 have at least one supporting EST (Table 3 in S1 File). In total 25 of the 31 physical gene clusters have some level of EST support. The consecutive genes SS1G_00891 and SS1G_00892 in cluster C012 are both annotated as being endoglucanases, contain the carbohydrate binding module (PF00734), and have EST support in the tomato infection library suggesting they may be co-ordinately regulated under these infection conditions. Six other gene clusters had EST support in the same library suggesting that under the same environmental conditions and/ or during a specific physiological process (Table 3 in S1 File). In comparison, the predicted genes SS1G_01426 and SS1G_01428 from gene cluster C002 had very strong EST support in the developing apothecia library (G787) associating these proteins with this process rather than plant infection.

## Discussion

The identification and analysis of the secretome is an essential tool in the investigation of how a fungal pathogen promotes infection. Full genome comparisons of fungi commonly overstate the potential secretome of the respective species through their reliance on signal peptide predictions. However, the use of a more stringent and identical bioinformatic pipeline, as described here for *S*. *sclerotiorum* and *B*. *cinerea*, and in previous studies of *F*. *graminearum* and *Z*. *tritici* [27–29], facilitates comparative analyses, while expediting the selection of putative secreted proteins for further investigation. The full genome comparison of *S*. *sclerotiorum* and *B*. *cinerea*, elucidated to the role of secreted CAZymes and peptidases in pathogenesis, yet failed to provide a deep analysis of the hundreds of additional secreted proteins. The recent Guyon *et al*. study [26] focused the characterisation of 486 *in planta* expressed *S*. *sclerotiorum* secreted proteins, identifying 70 *S*. *sclerotiorum*-specific candidate effectors. This secretome prediction utilised a less stringent selection criteria, with only 250/486 proteins being retained in the refined secretome, and was based on a limited interspecies comparison, where only 43/70 proposed *S*. *sclerotiorum* specific candidate effectors were retained in the refined secretome, yet none were shown to be *S*. *sclerotiorum* specific. In addition, the Guyon *et al*. secretome potentially excluded the involvement of the secretome in apothecia and/or sclerotia development. Hence, the present study defined the refined *S*. *sclerotiorum* and *B*. *cinerea* secretomes, including *in planta* and developmentally regulated genes. Comparative analyse revealed the shared and unique properties of the respective secretomes, while identifying novel candidate effectors. In addition, the wide ranging 115 interspecies comparisons enabled the identification of 30 secreted proteins specific to both *S*. *sclerotiorum* and *B*. *cinerea*, plus 11 *S*. *sclerotiorum*-specific and 32 *B*. *cinerea*-specific secreted proteins. Therefore, the present detailed analyses of the secretome of these two closely related necrotrophic fungi will permit more informed choices to be made when selecting candidate proteins to be tested for function during infection and/or fungal apothecia/ sclerotia development. Finally, the previous *S*. *sclerotiorum* secretome study by Guyon *et al*. did not explore the genomic landscape within which the predicted encoding genes resided. Within the current study, a number of tightly clustered genes coding for small secreted proteins were identified throughout the genome.

A refined secretome size of 432 *S*. *sclerotiorum* and 499 *B*. *cinerea* proteins was predicted using the identical bioinformatics pipeline described for wheat infecting pathogens *F*. *graminearum* and *Z*. *tritici*. The refined *S*. *sclerotiorum* and *B*. *cinerea* secretomes account for ~3% of their total predicted proteomes, which is relatively low compared with the representation of the secretome in *F*. *graminearum* (574 genes, 4.1%) and *Z*. *tritici* (492 genes, 4.46%) genomes [28,29]. The *S*. *sclerotiorum* secretome-encoding genes were evenly distributed across the 16 chromosomes whilst several small gene clusters were found that contained no more than 3 genes. These *S*. *sclerotiorum* small secretome gene clusters were similar to the *F*. *graminearum* small secretome gene clusters and dissimilar to the larger secretome gene clusters (ranging from 3 to 26 genes) in the *U*. *maydis* genome, which are known to be required for pathogenicity [28,58]. The *S*. *sclerotiorum* clusters did not contain any duplicated genes and only two clusters contained genes coding for similar protein families (C012 and C017). In accordance, the functionally related MCL clusters [26] where shown to not reside in physical gene clusters. The documented secreted virulence proteins SsCUTA, SSITL1, Sspg1 and Ssv263 were not found in these gene clusters. Interestingly, two of the LysM domain containing proteins residue very close to one another on chromosome 6 and are only separated by a gene annotated as a chitinase (SS1G_12510). This three gene cluster merits further investigation to determine whether these proteins perform similar functions to the known LysM effectors of other plant pathogens, for example, *C*. *fulvum* CfECP6, *M*. *oryzae* Slp1 and *Z*. *tritici* Mg3LysM [2,4,5].

Comparatively, the proportion of the functionally annotated secretome in *S*. *sclerotiorum* (310 genes, 71.8%) and *B*. *cinerea* (328 genes, 65.7%) was similar to *Z*. *tritic* (321 genes, 65.2%), while *F*. *graminearum* (278 genes, 48.4%) possessed a higher proportion of unannotated proteins. Unsurprisingly, both the *S*. *sclerotiorum* and *B*. *cinerea* secretomes contained a large battery of hydrolysing enzymes which degrade different plant host substrates. The repertoire hydrolytic enzymes in the respective secretomes were comparable in *S*. *sclerotiorum*, *B*. *cinerea* and *F*. *graminearum*, and smaller in *Z*. *tritici* [28,29]. Despite possessing a similar hydrolytic potential, *F*. *graminearum* exhibits a much smaller host range, predominantly infecting cereals. As suggested in previous studies [25], many of these polygalcturonases and proteases in *S*. *sclerotiorum* have optimum activities at an acidic pH, fitting with the secretion of oxalic acid ahead of fungal hyphal invasion [13]. The polygalacturonase gene family is the largest family of polysaccharide degrading proteins within the *S*. *sclerotiorum* secretome, whereas, in *B*. *cinerea* the carboxylesterases are the most expanded gene family. This again highlights some potential differences that may underlie the subtle differences in the infection strategies deployed by these two fungal necrotrophic species.

The functional analysis of the refined *S*. *sclerotiorum* and *B*. *cinerea* secretomes revealed a large number of proteins predicted to be involved in oxidation-reduction interactions, representing oxidases involved in multiple biological process including melanin production (tyrosinase) [35], lignin oxidation (laccases) [37] and isoamyl alcohol oxidase [59]. This is a striking contrast to the *F*. *graminearum* and *Z*. *tritici* secretomes. The redox state of the host substrate has been shown to be extremely important in determining the ability of *S*. *sclerotiorum* and *B*. *cinerea* to cause disease as demonstrated by the role of oxalic acid and reactive oxygen species, which are produced during infection to suppress the host plant oxidative burst and later to induce plant cell death [12,13,18,19]. The enzyme GMC oxidoreductase, also known as chloroperoxidases, accounts for ten members of the refined *S*. *sclerotiorum* and *B*. *cinerea* secretomes and would also be anticipated to influence the host or fungal redox balance. This enzyme has already been implicated in the biocontrol of other fungal species such as *F*. *oxysporum* [60]. Nine copies of the chloroperoxidase gene were predicted in the refined secretome of *Z*. *tritici* and were found to be prevalent in many other plant pathogens belonging to the Dothidiomycetes family [29]. Two oxalate decarboxylase enzymes are predicted in both *S*. *sclerotiorum* and *B*. *cinerea*. These enzymes may prevent the direct toxicity of oxalic acid to the fungus by hydrolysing excess oxalic acid. This would then allow a local rise in pH, which when sensed by hyphae, could then signal the downstream regulation of other genes involved in later infection processes, for example sclerotia formation.

The refined *S*. *sclerotiorum* and *B*. *cinerea* secretomes were explored for small, cysteine-rich proteins and proteins containing RXLR-dEER or Y/F/WxC motifs, which are characteristics of known effectors [1]. The representation of small (<200 amino acids) cysteine-rich (>5%) proteins in the *S*. *sclerotiorum* (22 genes, 5.1%) and *B*. *cinerea* (26 genes, 5.2%) secretomes was significantly lower than that of *F*. *graminearum* (61 genes, 10.6%) and *Z*. *tritici* (70 genes, 14.2%) potentially reflecting the diversification of this protein class in hemibiotrophic ascomycetes. Nonetheless, in *S*. *sclerotiorum* several of the unannotated cysteine-rich proteins had good *in planta* EST support. This included a small CFEM domain-containing protein that had strong EST support in the infected plant libraries and was highly induced during the microarray analysis of sunflower infection. CFEM domains have previously been implicated in plant pathogenesis [56]. The search for RXLR-dEER or Y/F/WxC motifs within the refined secretomes revealed both species to possess dioxygenase and proteolytic enzymes with a RXLR motif plus glycoside hydrolases with a Y/F/WxC motif. None of the *S*. *sclerotiorum* motif-containing candidate effectors were cysteine-rich proteins. This is in contrast to *B*. *cinerea*, which possessed four, and *Z*. *tritici*, which possessed 10, cysteine-rich proteins < 250 amino acids in length that contained a Y/F/WxC motif. Interestingly, the SS1G_02025 and BC1G_14481 proteins, which contained the Y/F/WxC motif, had good homology to a candidate effector 5 protein that has been found in two other fungal plant pathogens, namely the apple scab fungus *Venturia inaequalis* [49] and the grapevine dieback fungus *Eutypa lata* [50].

The multispecies comparison confirmed that the *B*. *cinerea* proteome shared the closest homology to the *S*. *sclerotiorum* secretome across all 432 proteins. This was an expected result because the similarity between the two species and their lifestyles is well documented [25]. The ~8% of secreted proteins which were different between the two refined secretomes are particularly interesting and may possibly contribute to the slightly different infection strategies adopted by these fungi. For example, *S*. *sclerotiorum* primarily secretes oxalic acid in very high levels during infection whereas *B*. *cinerea* secretes oxalic acid [61] as well as botcinolide, a highly substituted lactone [62] and botrydial, a tricyclic sesquiterpene [20]. In this study, *B*. *cinerea* was shown to have a reduced number of, or was lacking, chitin-binding, LysM, CFEM and metallophophoesterase domain containing proteins, while the *S*. *sclerotiorum* secretome possessed multiple copies. In a recent study, *B*. *cinerea* was shown to secrete small RNAs that subsequently enter the plant cells and inhibit the expression of specific target genes [24]. During *S*. *sclerotiorum* infection of plant tissue a role for small RNAs has not been reported. Therefore, the two closely related necrotrophs may adopt different mechanisms to influences the host plant cell.

Additionally, the multispecies analysis identified 30 proteins specific to *S*. *sclerotiorum* and *B*. *cinerea*, whereas no secreted proteins were found to be specific to a small group of fungal pathogens that could infect oilseed rape or amongst a larger group of ascomycete plant pathogens of dicotyledonous plants. This finding is unlike the result reported for the analysis of the refined *Z*. *tritici* secretome in which nine proteins were identified as being specific to fungal pathogens infecting wheat (*Triticum spp*) or other cereals [29]. One trivial explanation could be that not enough oilseed rape or dicotyledonous infecting plant pathogens were compared within this study. A second explanation could be the considerable host diversification of the plant pathogens compared in this study. Many of the dicotyledonous plant infecting fungal pathogens investigated have multispecies host ranges and therefore may require a general set of secreted proteins for infection, which utilise a highly conserved mechanism to promote disease. According to the tree of life (http://tolweb.org/angiosperm), monocotyledonous plants are grouped as a single taxon of Angiosperms whereas the plants we now know as Dicotyledonous are classified across a range of different taxa. It is tempting to speculate that fungal pathogens with a wider host range would require a larger and more diverse arsenal of secreted proteins, some of which would be conserved or overlap with pathogens with more specific host ranges. This may explain why in this study the proteome of *B*. *dothidea*, a plant pathogen infecting woody dicotyledonous species, was found to share such a large set of proteins with the secretome of *S*. *sclerotiorum*.

The analysis of the seven *S*. *sclerotiorum* EST libraries was extremely valuable when trying to assign some form of function to the unannotated putative secreted proteins. EST support was available for 57% of the putative proteins within the *S*. *sclerotiorum* secretome in a range of infection conditions and developmental stages. Similarly, a microarray analysis of *S*. *sclerotiorum* grown *in vitro* compared with sunflower cotyledon infection also provided transcriptional support for 57% of the secretome. Two secreted proteome studies, interrogating *in vitro* and sclerotia development extracellular fluids, confirmed the physical secretion of 42 proteins identified in the refined *S*. *sclerotiorum* secretome, which is of significant importance when validating the bioinformatics pipeline. The other secreted proteins which were not identified in these studies may be expressed during different conditions compared to those investigated via proteomics. For example, some protein secretion will only be induced during specific *in planta* infection conditions or at an earlier stage of spore germination. In addition, in both proteome studies did not use the sequenced *S*. *sclerotiorum* strain which may have contributed to protein differences in the computational search.

In summary, this *in silico* characterisation of the refined *S*. *sclerotiorum* and *B*. *cinerea* secretomes has provided new insight into how secreted proteins, alongside the secreted metabolites, may contribute to the necrotrophic infection. Although no direct associations can be made, many candidate proteins have now been identified. This fundamental area of research is anticipated to expand with the identification and functional characterisation of novel types of effector proteins, which will ultimately lead to the discovery of new targets for disease intervention, while providing new options for the control of economically destructive fungal pathogens such as *S*. *sclerotiorum* and *B*. *cinerea*.

## Materials and Methods

### Bioinformatics

Both the *S*. *sclerotiorum* (WT1980) and *B*. *cinerea* (B05.10) genomes used in this investigation were downloaded from the Broad Institute. *Sclerotinia sclerotiorum* and *Botrytis cinerea* Sequencing Project, Broad Institute of Harvard and MIT, (http://www.broadinstitute.org/annotation/genome/sclerotinia_sclerotiorum/MultiHome.html and http://www.broadinstitute.org/annotation/genome/botrytis_cinerea/MultiHome.html). The *S*. *sclerotiorum* genome of the ‘1980’ strain (ATCC18683) and the *B cineria* B05.10 genome sequence were downloaded in 2012. The *S*. *sclerotiorum* and *B*. *cinerea* genomes were analysed using a previously described and published software pipeline [27–29]. Briefly, for the first stage, the total secretome was predicted using Bash shell, awk and python scripts on a PC running Red Hat Enterprise Linux 5.2. For the second stage of the analysis the refined secretome was defined and then analysed in depth. Then the interspecies distribution of the genes present within the entire refined secretome was explored further in 115 species.

#### Stage 1: Predicting the total secretome

The first stage of the analysis followed the previously described method [28,29], which is described again here for clarity. An automated pipeline based on the original secretome prediction procedure described by Mueller [27] using Bash shell, awk and python scripts on a PC running Red Hat Enterprise Linux 5.2.

Initially all proteins from downloaded genomes with a Target P Loc = S (TargetP v1.1; http://www.cbs.dtu.dk/cgi-bin/nph-sw_request?targetp) or a Signal P D-score = Y (SignalP v3.0; http://www.cbs.dtu.dk/cgi-bin/nph-sw_request?signalp) were combined [63,64] to predict the refined secretome. These were then scanned for transmembrane (TM) spanning regions using TMHMM (TMHMM v2.0; http://www.cbs.dtu.dk/cgi-bin/nph-sw_request?tmhmm) and all proteins with 0 TMs or 1 TM, if located in the predicted N-terminal signal peptide, were kept. GPI-anchor proteins were predicted by big-PI (http://mendel.imp.ac.at/gpi/cgi-bin/gpi_pred_fungi.cgi) [65]. ProtComp was also used to predict localisation of the remaining proteins using the LocDB and PotLocDB databases (ProtComp v8.0; http://www.softberry.com). WoLF PSORT analysis was done using ‘‘runWolfPsortSummaryfungi” in the WoLF PSORT v0.2 package, which estimates where proteins are located after secretion with a sensitivity and specificity of approximately 70% [66].

Additionally, PFAM analysis was done using the PFAM database (ftp://ftp.ncbi.nih.gov/pub/mmdb/cdd/) and the rpsblast program in the NCBI blast+ software package (ftp://ftp.ncbi.nlm.nih.gov/blast/executables/blast+/). http://pfam.sanger.ac.uk/ was also accessed for direct inspection of protein domains. The number of cysteine residues within the mature peptide and the search for degenerative YFWxC and RXLR motifs were computed using custom python scripts. The number of internal sequence repeats was found using RADAR (http://www.ebi.ac.uk/Tools/Radar/) [67]. The detection of RNA transcripts for the *S*. *sclerotiorum* genes of interest was explored by BLASTN analysis (e-100) of the 7 designated EST libraries available from the Broad website: (http://www.broadinstitute.org/annotation/genome/sclerotinia_sclerotiorum/MultiDownloads.html). All nucleotide and amino acid sequences were aligned in Geneious Pro5.5.6 created by Biomatters. Available from http://www.geneious.com/. ClustalW and MUSCLE alignments were used for the analysis.

#### Stage 2: The refined secretome

The second stage of the analysis generated the refined secretome for both species from which secreted proteins for both genomes could be identified. A comparison between the refined secretomes was made to explore the relatedness and evolution of the two pathogens. Initially only sequences starting with a methionine were selected. Then sequences predicted with an extracellular WolF-PSORT score of 18 and above were kept in the final secretome dataset. This selection ‘cut-off’ point has been tested using a range of experimentally verified secreted fungal proteins from other pathogens including *F*. *graminearum* [28] and *Z*. *tritici* [29]. Any mature proteins shorter than 20 amino acids were removed and sequences with 1TM were excluded from the refined set. A comparison between those sequences containing a PFAM domain were analysed and the protein family for each domain identified using http://pfam.sanger.ac.uk.

### Genes coding for proteins with a known function

Genes identified in the refined secretome with a PFAM domain were inspected for gene function and grouped into the four CAZy classes; glycoside hydrolases, glycosyl transferases, polysaccharide lyases and carbohydrate esterases listed on the Carbohydrate-Active EnZymes database (CAZy) (http://www.cazy.org/) [68]. Enzyme identification was carried out using the Kegg database (http://www.kegg.jp/) and Brenda (http://www.brenda-enzymes.info/).

### *S*. *sclerotiorum* genome map

A genome map for *S*. *sclerotiorum* was generated using the free software, OmniMapFree (http://www.omnimapfree.org) [69]. This allowed easy mapping of different gene groups to inspect visually the distribution of genes across the chromosomes.

### Blast2GO analyses

The protein set within the predicted refined secretome was analysed using Blast2GO to explore whether any additional gene annotation existed (http://www.blast2go.com/b2glaunch) [70]. From this tool, the InterPro website was accessed to further explore the IPO entries discovered http://www.ebi.ac.uk/interpro/ [71,72].

### EST and Microarray hybridization support

Seven *S*. *sclerotiorum* EST libraries were downloaded from the Broad Institute online database to determine whether there was any expression data to support the gene models of interest. The EST libraries were generated from different biological materials and culture conditions (Table 1). Microarray hybridization data obtained from the comparison of *in vitro* mycelia growth with sunflower cotyledon infection was downloaded from http://urgi.versailles.inra.fr/Data/Transcriptome. Transcripts with a corrected *p*-value <0.05 and more than 2.0 fold change in transcript level were considered as significantly differentially expressed.

### Multispecies comparison

A multispecies comparison was done between the refined secretome of *S*. *sclerotiorum* and the predicted gene calls of 115 species including pathogenic and non-pathogenic fungi and oomycete [28,29] plant pathogenic nematodes and plant infecting aphid species. The species were chosen based on the diverse range of lifestyles and host ranges. The gene calls from each genome were downloaded (November 2012) from the Broad, JGI and other websites which are used primarily by the research community for the species in question (Supplementary Table S3). Conservation, absence or expansion of the homologues genes from the total secretome for *S*. *sclerotiorum* and *B*. *cinerea* were found in the other species using BLASTP analysis. The levels of confidence used were p<e^-5^ or p<e^-100^. These multispecies comparisons allowed the identification of unique proteins in both species that were not found in any other species. These genes were then inspected for a cysteine number greater than 5, a Wolf-PSort score of 17 or greater, and no PFAM or other annotations.

## Supporting Information

S1 File The *Sclerotinia sclerotiorum* entire and refined secretome predictions. (XLSX)

S2 File The *Botrytis cinerea* entire and refined secretome predictions. (XLSX)

S3 File The 115 interspecies comparison of the refined *Sclerotinia sclerotiorum* and *Botrytis cinerea* secretomes. (XLSX)
